# Hook plate fixation of acute displaced lateral clavicle fractures: mid-term results and a brief literature overview

**DOI:** 10.1186/1749-799X-7-2

**Published:** 2012-01-11

**Authors:** Davut Tiren, Alexander JM van Bemmel, Dingeman J Swank, Frits M van der Linden

**Affiliations:** 1 Department of Surgery, Amphia Ziekenhuis, Breda, The Netherlands; 2 Department of Surgery, Groene Hart Ziekenhuis, Gouda, The Netherlands

## Abstract

**Background:**

The clavicle hook plate achieves like most other operative techniques, a high percentage of union and a low percentage of complications however concerns about long term complications still exist, particularly the involvement of the acromioclavicular joint.

**Methods:**

To evaluate the results and long term effects in use of this plate we performed a retrospective analysis with a mean follow up of 65 months (5.4 years) of 28 consecutive patients with acute displaced lateral clavicle fractures, treated with the clavicle hook plate.

**Results:**

Short term functional results in all patients were good to excellent. All but one patient had a united fracture (96%). Nine patients (32%) developed impingement symptoms and in 7 patients (25%) subacromial osteolysis was found. These findings resolved after plate removal. Twenty-four patients were re-evaluated at a mean follow-up period of 5.4 years. The Constant-Murley score was 97 and the DASH score was 3.5. Four patients (14%) developed acromioclavicular joint arthrosis of which one was symptomatic. Three patients (11%) had extra articular ossifications of which one was symptomatic. There was no relation between the impingement symptoms, subacromial osteolysis and development of acromioclavicular joint arthrosis or extra articular ossifications.

**Conclusions:**

The clavicle hook plate is a good primary treatment option for the acute displaced lateral clavicle fracture with few complications. At mid term the results are excellent and no long term complications can be addressed to the use of the plate.

## Background

In the last decade, the clavicle hook plate has been used extensively [1-10]. Although this plate achieves, like most other operative techniques, a high percentage of union and a low percentage of complications, concerns about long term complications still exist, particularly the involvement of the acromioclavicular joint (ACJ) [11].

To evaluate the results and long term effects in use of this plate we performed a retrospective analysis with a mean follow up of 65 months (5.4 years) of 28 consecutive patients with acute displaced lateral clavicle fractures, treated with the clavicle hook plate.

## Methods

All patients diagnosed with a displaced lateral clavicle fracture in our hospital from 2001 to 2008 were retrospectively assessed.

Two experienced trauma surgeons operated on these patients. Unrestricted passive and active range of motion was performed as soon as possible after the operation. Clinical and radiological union was assessed after which patients underwent plate removal.

The clinical files were analyzed and the x-rays re-evaluated. After initial analysis, all patients were reassessed at the outpatient clinic. After informed consent, objective and subjective shoulder function evaluation was performed with the DASH and Constant-Murley scoring systems after which patients were radiographically assessed.

No statistical analysis was performed.

### The Implant

The clavicle hook plate used in this study is a pre-contoured stainless steel, dynamic compression plate with a wider anterolateral end and a lateral extension into a hook which is placed below the acromion. The holes accept 3.5 mm cortical bone screws and 4.0 mm cancellous bone screws. The anterolateral screw holes provide additional options for screw fixation of the lateral metaphyseal part of the clavicle. These plates are available with 6 or 8 holes and the hook depth is variable between 15 and 18 mm's.

### Surgical Technique

Our surgical technique consisted of application of basic reduction and plating methods, following the operative procedure as advised by the 'Synthes clavicle hook plate - technique guide' (2003 Synthes).

The patients were operated in beach chair position under general anaesthesia with the arm on the affected side, freely moveable. A sagittal incision was placed just medial to the acromioclavicular joint over the fracture. Full thickness skin flaps were prepared until the clavicle. The fracture was reduced; large comminuted fragments were temporarily fixed with K-wires and sometimes a lag screw was used. No repair of the torn ligaments was performed. Any interposed tissue was removed. Without opening the AC joint, the location of the joint was marked with a needle, and confirmed with fluoroscopy. The soft tissue dorsal to the AC joint was dissected and prepared for the insertion of the hook of the plate. First the 15 mm hook depth was used and passed below the acromion. The shaft of the plate was placed on the superior aspect of the clavicle and checked for alignment. No excessive levering with the plate was performed to reduce the fracture. In case of difficulty lowering the plate shaft onto the clavicle, the hook depth of 18 mm was used. If excessive force or torque was needed, the reduction was verified and if needed altered. The clavicle portion of the plate was slightly bent to ensure central placement of the plate on the clavicle. The tip or hook portions were never bent. Before definitive fixation, plate position and full shoulder motion was verified using fluoroscopy. The plate was then secured to the shaft with four 3.5 mm cortical screws approximating the plate to the clavicle. If necessary, the distal metaphyseal end was secured to the plate through the anterolateral holes with cancellous screws. In patients with osteoporotic bone, an 8 hole plate was used. The wound was closed in layers over the plate.

## Results

### Demographics

All twenty-eight patients diagnosed with a displaced lateral clavicle fracture between 2001 and 2008, were treated with the clavicle hook plate. Mean age was 38 years (range 15-64), male to female ratio was 21 to 7. Fourteen patients had a right sided and fourteen a left sided fracture. All patients had an Edinburgh Type 3B1/Neer Type II fracture. All patients had suffered a monotrauma. Mean time to operation was 5 days (range 0-14 days) and the operating time was 43 minutes (23-70 minutes). All patients were discharged on the day of or the day after operation. After a mean follow up of 6 months (range 2-14 months), the plate was removed under general anaesthesia. Short term follow up of patients ended after a mean period of 7 months (range 3-13 months) starting from the initial operation.

Mid term follow up was from 15 to 103 months with a mean of 65 months (5.4 years). Five patients were lost to follow up. One patient had been a victim of a traffic accident. Two patients could not be traced and two other patients refused to participate in the study.

### Short term results and complications [Table 1]

**Figure 1:**
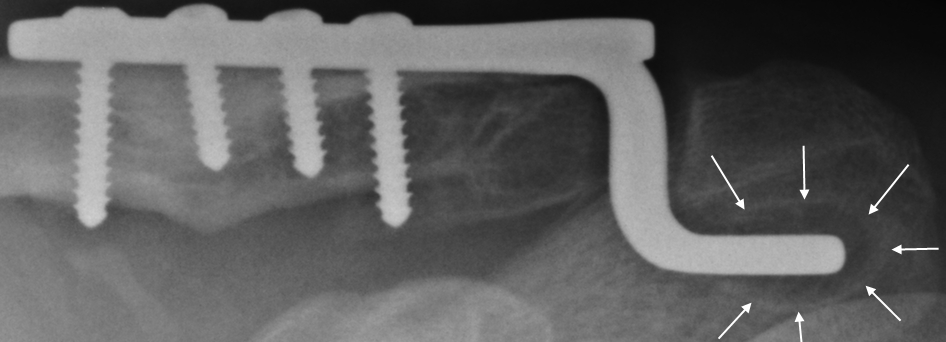
**Lucency around the tip of the hook (subacromial osteolysis)**.

During the out-patient clinic follow up ten patients reported pain. Nine of these patients were diagnosed with impingement and this resolved shortly after plate removal. One patient's symptoms did not resolve: he was diagnosed with ACJ arthrosis and had to undergo a lateral clavicle resection for relief of symptoms. In 7 patients lucency around the tip of the plate was noted, radiologically diagnosed as subacromial osteolysis [Figure 1]. Four of these patients also had impingement complaints. After plate removal, the osteolysis disappeared on follow up radiographs.

One patient was diagnosed with a non union due to a misplaced hook of the plate. This patient developed an asymptomatic non union with a good alignment of the fracture, probably due to fibrous alignment of the ligaments.

**Table 1:** patient characteristics, findings during initial and final follow up

Nr	Side	Age	Sex (M/F)	Operation Date	Time to Surgery (days)	Operating time (min)	Complications	Reason plate removal	Time to removal (mo)	Initial Followup (mo)	Final Followup (Years)	Constant	DASH	x-Ray
1	R	64	M	2001	7	33		Routine	14	5	8,5	100	0	
2	R	18	M	2001	4	70		Impingement	10	11	8,3	100	0	SAO
3	R	52	F	2001	1	50		Routine	11	12	8,3	90	5	
4	R	40	M	2002	6	28		Routine	8	9	8,1	100	1,6	ACJ arthrosis
5	R	30	M	2002	10	52			Not removed	10	LOST			
6	L	48	M	2002	3	51		Impingement	4	4	7,7	100	0	SAO
7	R	32	F	2002	3	55		Impingement	3	7	7,5	99	0	
8	L	19	F	2002	1	41		Routine	8	8	7,3	100	0	SAO
9	L	52	F	2003	0	50		Routine	7	8	7,2	100	0	
10	L	52	M	2003	7	55		Routine	7	5	6,7	79	14,2	EAO
11	R	44	M	2003	0	40	ACJ arthrosis	Pain	4	4	6,7	100	25	Lateral clavicle resection
12	L	34	M	2003	2	55		Routine	4	4	6,5	100	0	
13	L	52	M	2004	5	51		Impingement	6	7	6,1	100	0	
14	R	28	M	2004	1	40		Impingement	11	13	6	100	0	SAO
15	R	44	M	2004	5	30		Routine	5	6	LOST			
16	R	28	M	2005	14	50		Routine	8	10	5	100	7,5	
17	R	15	M	2005	3	43		Routine	7	7	5	100	0	
18	R	17	M	2005	4	54		Routine	4	12	4,9	100	1,6	ACJ arthrosis
19	L	36	M	2005	10	52	Wound infection	Routine	3	5	LOST			SAO
20	L	25	M	2005	6	28		Routine	6	7	LOST			SAO
21	L	49	M	2006	6	23		Impingement	5	7	3,4	100	1,6	ACJ arthrosis
22	L	64	M	2006	11	51		Impingement	2	10	3,3	68	8,3	Poliomyelitis, EAO
23	L	29	M	2007	6	38		Routine	3	4	2,9	100	1,6	
24	R	61	F	2007	0	31	Non union	Routine	5	6	2,6	100	0	Non union
25	L	36	F	2008	12	39		Impingement	2	3	LOST			
26	L	18	M	2008	1	29		Impingement	3	4	1,8	100	0	SAO
27	L	44	F	2008	2	42		Routine	3	5	1,4	100	13,3	
28	R	25	M	2008	0	35		Routine	4	10	1,3	100	0	

Mean		38			5	43			6	7	5	97,2	3,5	

ACJ = acromioclavicular joint; EAO = extra articular ossification; SAO = Subacromial osteolysis; M = Male; F = Female; R = Right; L = Left

One patient developed a superficial wound infection that was treated successfully with oral antibiotics. The plate was removed as soon as possible after union.

All patients were advised to remove the plate after clinical and radiological consolidation. Twenty-seven of the 28 patients were operated upon for plate removal. One patient refused plate removal, because of lack of complaints. There were no peri - or postoperative complications.

Subjectively, all patients described their shoulder function as good to excellent at the moment of discharge from the outpatient clinic.

### Mid term results and complications [Table 1]

The mean Constant-Murley score was 97 (68-100) and the mean DASH score was 3.5 (0-25). The lowest Constant Murley score (68) was of a patient who had suffered from poliomyelitis on the involved side and had returned to the same subjective function as before the fracture. The highest DASH score (25) was from the patient with the lateral clavicle resection due to the symptomatic ACJ arthrosis. Previously observed union of the fracture and the non union in one patient was confirmed radiographically. In three patients ACJ arthrosis was observed. These patients had no symptoms, although their DASH scores were 1.6. Only one of these patients with ACJ arthrosis had suffered impingement symptoms while the plate was in situ, without any evidence of subacromial osteolysis on the radiographs. In three patients extra articular ossification was noted. Only one patient was symptomatic with a lower Constant score (79) and a higher DASH score (14).

## Discussion

The displaced lateral clavicle fracture is an uncommon fracture. Although 15% of all clavicle fractures consist of lateral clavicle fractures, only a third of these fractures are displaced (Neer Type 2/Edinburgh Type 3B1)[12].

Due to the rarity of this fracture, literature consists mainly of retrospective case series with small number of patients, some with inclusion of heterogeneous patient population, usually with a short and sometimes incomplete follow up.

Neer described this type of clavicle fracture as an unstable clavicle fracture requiring operative treatment due to the high rate of observed non union and the even higher rate of delayed union. He explained this by the deforming forces around the fracture, causing displacement and interpositioning between the fracture fragments, with continuous motion at the fracture ends [13-15].

### Treatment of the displaced lateral clavicle fracture in the literature

Conservative management has been advocated by several authors. Rokito et al [15] retrospectively compared results of 16 conservatively and 14 operatively treated patients with displaced lateral clavicle fractures. They reported a high percentage of non union in the conservatively treated group (7/16) while the shoulder function was comparable in both groups after approximately 4.5 years. Robinson and Cairns [16], retrospectively followed up on 101 patients. According to their policy, the treatment was conservative during the first six months. If still symptomatic after six months, patients were treated operatively. They reported a non union of 37%. Only 35% of these patients required an operation because of symptoms. Only 14 of the 101 (14%) patients were operated on because of persisting symptoms after 6 months. The functional results at follow up of the different groups were similar.

Operative treatment of these fractures can be a challenge because of the small and soft metaphyseal and usually comminuted distal fragment and the proximity to the AC joint. Several methods have been described.

Transacromial wire fixation was popularized by Neer [14] and is a commonly used method. Kona et al [17] reported an unacceptably high complication rate (47%) with the use of K-wires and advised against its use. Flinkkila et al [1] compared K-wire fixation to hook plate fixation. Although the functional results were similar, they advised hook plates because of migration and infection in the K-wire group. Lee et al [2] compared K-wire fixation with tension band wiring to hook plate fixation. Their results showed that the group with the hook plate had earlier regain of pre-injury activities. The K-wire fixation group had 30% complications related to hardware failure.

Another operative treatment option is indirectly reducing the fracture by coracoclavicular fixation. Using this method, several techniques have been described. Ballmer and Yamaguchi reported good results with the Bosworth screw fixation [18,19]. Similarly several methods have been described where a PDS suture, a Dacron patch or an Endobutton^© ^device through bore holes is used to perform the fixation [20-22]. The indirect reduction method requires extensive dissection around the fracture and bore holes through the clavicle and the coracoid process. Erosion of these structures and fracture of the clavicle and the coracoid are well recognized complications [17,23,24]. Especially in case of the rigid fixation with the Bosworth screw, and in lesser extent with the other devices, the rotation of the clavicle is disabled requiring partial immobilization of the shoulder until fracture consolidation with the potential of implant breakage and a longer revalidation period.

Despite the small, soft and sometimes comminuted metaphyseal fragment, Regazzoni et al [11] described extra articular double plating of this fracture, using mini AO plates with similar results and complications to other operative treatments.

### Treatment with the clavicle hook plate

The clavicle hook plate is an easy to handle solid plate that withstands forces that are applied to the fracture fragments. By design it keeps the lateral end of the clavicle reduced, hereby aligning the clavicle with the ligaments and minimizing movement at the fracture ends while it does not interfere with the rotational movement of the clavicle [25]. The results published in several studies [1-10] show good results in terms of bony union and in terms of shoulder function. Shoulder function is measured most frequently by the DASH and Constant-Murley scores. The DASH score is usually below 5 and the Constant-Murley score averages around 90. Non union occurs only seldom, below 10% in most series. Compared to the K-wire fixation and the Bosworth screw fixation, it facilitates earlier regain of previous activities [1,2,24].

### Complications of the clavicle hook plate

Although the types of fractures included, mean follow up time, postoperative mobilization and plate removal policy varies in different publications, several typical complications are associated with the hook plate.

The first category is related to the freely movable hook of the plate that is placed posterior to the AC joint, below the acromion, and above the supraspinatus tendon. Even though the design of the hook plate promotes fracture healing by keeping the fracture fragments reduced without interfering with the rotational movement of the clavicle, this design also leads to complaints due to mismatch between the hook of the plate and the diverse anatomy of the acromion.

El Maraghy et al [26] demonstrated the mismatch between the plate and the subacromial space leading to several well described short term complications in an anatomic study. In 89% of the specimens the hook perforated the subacromial bursa, in 60% the tip had contact with the supraspinatus tendon and in 60% contact with the acromion was concentrated at the tip of the plate. These findings clarify the subacromial bursitis, the impingement complaints and the subacromial osteolysis respectively. They concluded that the anatomy of the acromion is too diverse to accommodate a single hook plate and when necessary the hook and the tip of the plate needs bending and smaller depths of the hook should be selected if necessary, especially for women.

Lee et al [10] performed arthroscopy during the procedure to verify the position and fit of the hook and tip besides intra-operative fluoroscopy verification. If necessary the tip and the plate was bent according to the required anatomy of the patient. They also had access to the new LCP plate which comes in a smaller depth of 12 mm. In this case series none of the patients suffered impingement. However they still encountered subacromial osteolysis (17%) and subacromial bursitis (22%).

Muramatsu et al [8] found it necessary to bend the hook in 77% of their patients, and found in most of their patients, migration of the hook after fixation. Their operative technique describes however, forcefully reducing the fracture using the plate as a lever.

Impingement, subacromial bursitis and subacromial osteolysis on x-ray are signs of a mismatch between the plate and the anatomy of the patient. These complications can be minimized by performing an anatomic fit of the plate during the procedure.

However, the plate design is such, that the vertical part of the hook and the tip must have contact with the underside of the acromion hereby maintaining reduction of the fracture and withstanding forces applied to the fracture ends. Pressure concentration at the tip of the plate that leads to subacromial erosion due to the rotation of the clavicle when the implant is retained for a longer period, becomes unavoidable in part of the patients. Similarly, contact with the supraspinatus tendon in some cases is unavoidable, even though there is no contact during the operation, the contact may happen when abducting the arm during the rehabilitation period.

Even though aforementioned short term complications have the potential of acromion fracture, and supraspinatus tendon rupture, these complications have never been reported with this plate in the literature [8].

In our patient group, we used the surgical technique as described above. We had impingement complaints in 32% and subacromial osteolysis in 25% of our patients [Figure 1]. These complaints were mild and all patients could complete their rehabilitation program. None of these patients developed a frozen shoulder or required early plate removal. The impingement complaints as well as subacromial osteolysis resolved after plate removal and had no mid term consequences.

Another complication is a fracture medial to the plate that can be seen with a minimal trauma. This complication has only been described with a retained implant after fracture healing [27,28].

The last category of complications are typical complications of plate osteosynthesis such as fixation failure due to osteoporotic bone and deep infection of the plate [27-29].

Several long term complications associated to the lateral clavicle fracture have also been described in relation to the use of this plate. These are ACJ arthrosis and extra articular ossifications. Due to the proximity of this plate to the ACJ, several authors discourage use of this plate [11,22]. When placed correctly, the plate does not violate the ACJ. However the vertical part of the hook passes behind the ACJ. This part of the plate could violate the joint if the plate migrates anteriorly but this is almost impossible when secured rigidly on the shaft.

ACJ arthrosis and extra articular ossification have been described in all types of lateral clavicle fractures in studies where there was longer term follow up.

Nordqvist et al. [30] described a cohort of conservatively treated lateral clavicle fractures with a mean follow up of 15 years. They reported 7 ACJ arthrosis in 89 patients. Five of these occurred after a type I fracture, 1 after a type2 and 1 after a type 3 fracture. Extra articular ossification was observed in 8 cases. Robinson et al [12,16] described a prevalence of 9% up to 15% of ACJ arthrosis in patients with conservatively treated lateral clavicle fractures. Flinkkila et al [5] described 63 patients with displaced lateral clavicle fractures treated with the clavicle hook plate. Fifty percent of the patients were clinically re-evaluated with a mean follow up of 3.6 years. Ten of 31 followed up patients (32%) had mild asymptomatic ACJ arthrosis.

We analysed our patient population to find a relation between occurrence of ACJ arthrosis and extra articular ossification detected at mid term follow up and and signs of a mismatch between the plate and the subacromial space such as impingement and subacromial osteolysis.

**Figure 2:**
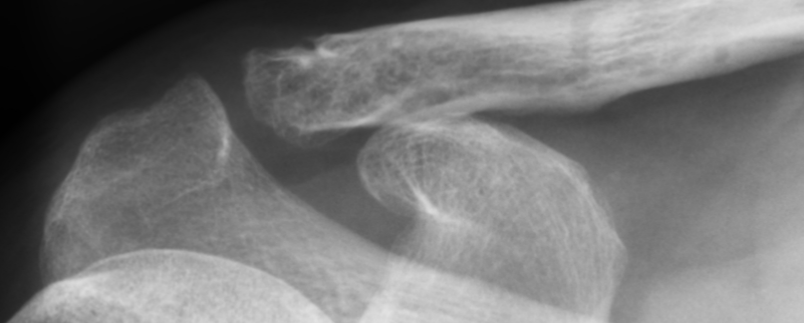
**Mild asymptomatic ACJ arthrosis**.

**Figure 3:**
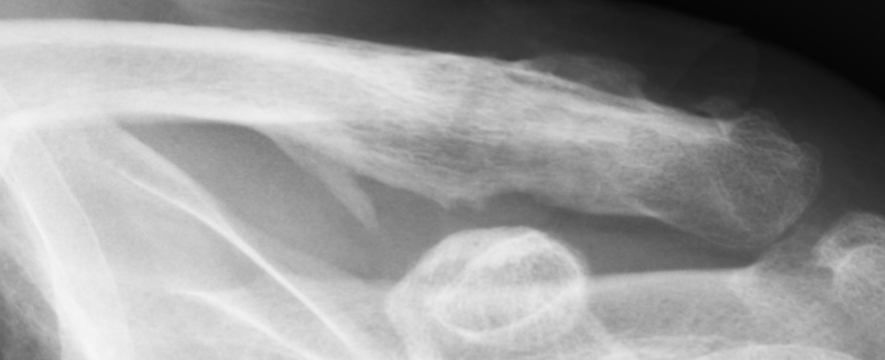
**Extra articular ossification**.

In our study, 4 patients (14%) had ACJ arthrosis [Figure 2], of which one was symptomatic. Only one patient with ACJ arthrosis had suffered impingement without signs of subacromial osteolysis. Three patients (11%) had extra articular ossification [Figure 3] of which one was symptomatic. Only one of the patients with extra articular ossification had suffered impingement and had no signs of subacromial osteolysis.

Even though the numbers are small to perform statistical analysis, we found no relation between ACJ arthrosis, extra articular ossifications at mid term follow up and the typical short term complications occurring due to mismatch of the plate tip and the acromion. In light of previous publications [5,12,16,30] about the lateral clavicle fracture, ACJ arthrosis as well as extra articular ossification is more likely to be caused by the initial trauma to the joint and the ligaments rather than a complication that can be addressed to the hook plate.

The strength of this study is in its high rate of follow up duration, the uniformity of the included fractures and the number of included patients for such a rare fracture. To our knowledge, this study has the longest mean time of follow up in the literature concerning primary operative treatment of acutely displaced lateral clavicle fractures with the clavicle hook plate. Our study is retrospective with limitations of this design. Even though we operated on all displaced lateral clavicle fractures, a possible selection bias is the age of our patient population since our series is younger than some described series. Younger patients have fewer complications due to better bone quality and better circulation of tissues which could explain the low percentage of infection and the high percentage of union in our report.

## Conclusion

Operative treatment of patients with displaced lateral clavicle fractures with the hook plate has produced good short term as well as mid term results. Using this plate may cause impingement and subacromial osteolysis, without leading to functional impairment. These complications can be minimized by meticulously adjusting the plate to the individual anatomy with verification under fluoroscopy and/or arthroscopy. A second operation is needed to remove the plate after fracture consolidation. In the short term follow up after plate removal, impingement complaints and the osteolysis disappear. In this study we found no relation between these short term complications and mid term functional results.

We conclude that clavicle hook plate fixation is a good primary treatment for the displaced lateral clavicle fracture. It facilitates early mobilization of the shoulder postoperatively and results in a high percentage of union with a good objective and subjective shoulder function. Part of the treated patients do develop impingement symptoms due to a mismatch between the plate and patient anatomy, one of the reasons the plate has to be removed after fracture consolidation. Mid term follow up shows no additional damage done to the surrounding structures that can be addressed to the use of this plate.

## Competing interests

No external financial support was received in support of this study.

## Authors' contributions

DT and AJMB designed the study. FML and DJS operated on the patients and performed the short term follow up. AJMB and DT performed the mid term follow up. DT prepared the manuscript and revisions. All authors read and approved the final manuscript.
